# Promoting Holistic Student Development Through Universal School-Based Social-Emotional Learning in China: A Meta-Analysis

**DOI:** 10.3390/bs16030368

**Published:** 2026-03-05

**Authors:** Zheyu Lin, Chunhan Huang, Jieping Shi

**Affiliations:** 1 Faculty of Education, Beijing Normal University, Beijing 100875, China; linzheyu@mail.bnu.edu.cn; 2 College of Life and Environmental Sciences, University of Birmingham, Birmingham B15 2TT, UK; cxh1094@student.bham.ac.uk

**Keywords:** social-emotional learning, meta-analysis, Chinese education, school-based intervention

## Abstract

Social-emotional learning (SEL) is a crucial pathway for cultivating students’ social-emotional skills and promoting their holistic development. This study presents the first meta-analysis of universal school-based SEL interventions implemented in the PreK-12 educational context of mainland China. Based on 52 studies involving 13,969 students, the analysis reveals a significant, medium overall effect (ES = 0.56), demonstrating the positive impact of school-based SEL programs on Chinese students’ development. However, given that the majority of included studies employed quasi-experimental designs with relatively small sample sizes, this effect should be interpreted as primarily reflecting efficacy under controlled conditions rather than effectiveness in real-world implementation contexts. Outcomes vary by domain, with the strongest effects observed in social-emotional skills (ES = 0.63), followed by academic performance (ES = 0.34), behaviors (ES = 0.31), and affect and attitudes (ES = 0.24). Moderator analyses further reveal that sample size, intervention dosage, implementer, and grade level significantly influenced effect sizes, whereas publication type, duration, source of outcome data, and intervention approach did not. Cross-cultural comparisons further contextualize these findings within the international SEL literature. By establishing an initial evidence base, this review highlights the promise of school-based SEL in China, while also noting limitations in external and ecological validity. Future research should prioritize more large-scale RCTs, focus on the quality of implementation, and address the needs of marginalized student groups.

## 1. Introduction

Social-emotional skills are widely recognized as fundamental to individual success and well-being. They contribute to improved academic achievement, career outcome, physical and mental health, and life satisfaction (Borghans et al., 2011; Duckworth & Seligman, 2005; Heckman et al., 2006; Strickhouser et al., 2017). Given that these skills can be effectively cultivated through education and training (Almlund et al., 2011), integrating their development into school systems has become a consensus among international organizations, policymakers, scholars, and educational stakeholders (Chernyshenko et al., 2018; Heckman & Kautz, 2012; Jennings & Greenberg, 2009). Consequently, schools worldwide are exploring effective strategies, with Social-Emotional Learning (SEL) established as a prominent, evidence-informed framework for such efforts.

SEL is defined as the process through which all young people and adults acquire and effectively apply the knowledge, attitudes, and skills necessary to understand and manage emotions, set and achieve positive goals, feel and show empathy for others, establish and maintain positive relationships, and make responsible decisions (Durlak et al., 2015). This study adopts this comprehensive definition, aligning with the conceptualization used in previous meta-analytic reviews such as that by Durlak et al. (2011). In practice, school-based SEL implementation has evolved from SEL curricula to systemic SEL reform models (Du & Mao, 2019). The curriculum model, rooted in emotional intelligence theory, delivers direct instruction but often becomes marginalized in school schedules and depends heavily on teacher capacity. Currently, the systemic reform model emphasizes integrating SEL into whole-school practice, where leadership support, family engagement, and teacher development are crucial for sustainable implementation (Osher et al., 2016).

In China, recent years have witnessed a proliferation of experimental studies investigating school-based SEL interventions. However, the findings regarding their impact on students remain inconsistent and inconclusive. To address this gap, the present study provides the first systematic synthesis and meta-analysis of universal school-based SEL interventions implemented in the PreK–12 educational context in mainland China. Employing an evidence-based research approach, this meta-analysis aims to synthesize the overall effects of Chinese school-based SEL programs on student outcomes, thereby offering rigorous and reliable evidence to guide evidence-based practice and educational decision-making.

### 1.1. Previous Relevant Reviews

Previous meta-analytic evidence suggests that universal school-based SEL interventions exert broadly positive effects on student development. For instance, Durlak et al. (2011), in their synthesis of 213 studies for K-12 students, found a significant positive effect (ES = 0.57), with follow-up effects remaining significant though smaller (Taylor et al., 2017; ES = 0.23). Sklad et al. (2012) reported the highest overall effect (ES = 0.70) across 75 studies involving primary and secondary students. Wigelsworth et al. (2016) observed a moderate effect (ES = 0.53) across 89 studies of students aged 4 to 18 years, while Van De Sande et al. (2019) reported effects ranging from 0.24 to 0.58 specifically in secondary schools. More recent reviews tend to report smaller effects; for example, Cipriano et al. (2023) reported an ES of 0.22, and Shi and Cheung (2024a) found an improvement of 0.17 standard deviations across 12 high-quality PreK-12 (students aged 3–18) programs.

Another set of reviews has focused specifically on preschool populations. Blewitt et al. (2018) evaluated 63 early childhood programs, reporting effect sizes of 0.30 for social competence, 0.54 for emotional competence, and 0.28 for self-regulation. Murano et al. (2020) identified a medium overall effect (ES = 0.34) across 33 studies, and W. Yang et al. (2019) observed a modest effect (ES = 0.24) among low-income preschoolers. In summary, while universal school-based SEL demonstrates consistent benefits, its effect sizes vary considerably, typically ranging from 0.17 to 0.70, indicating overall weak to moderate impacts.

Existing meta-analyses on school-based SEL interventions have predominantly drawn from English-language publications and studies conducted in Western contexts, leaving a significant gap regarding the effectiveness of such programs within China. This gap is particularly noteworthy given the ongoing debate surrounding the cross-cultural applicability of SEL frameworks (Chong & Lee, 2015), underscoring the need for a context-specific synthesis of evidence from China. There are only two meta-analyses that have examined SEL interventions in Chinese settings. One, by (H. Chen & Yu, 2022), reported that SEL interventions significantly improved social-emotional competence, with a mean effect size of 0.36. However, as this review included both school-based and non-school-based interventions, its findings cannot be directly translated into evidence-based practices for educational settings. Another relevant study by (Y. Yang et al., 2025) systematically reviewed mental health promotion and prevention programs for adolescents in Chinese societies and found a significant improvement in social-emotional competencies (e.g., self-esteem and self-efficacy), with a pooled effect size of 0.39. While informative, this review was not framed within an SEL-specific theoretical lens, limiting its comparability to the broader SEL literature. Therefore, this study aims to address these gaps by conducting a focused meta-analysis of school-based SEL interventions implemented in China. Furthermore, to provide testable implications for the cross-cultural applicability of SEL, this study will operationalize cultural adaptation as a moderator variable to compare the effects of indigenous versus Western-adapted interventions.

### 1.2. Chinese Context and SEL in China

Reflecting a global shift in educational priorities toward holistic development (OECD, 2015), China is increasingly integrating SEL into its educational system. However, implementing SEL, a framework largely developed in Western contexts, presents distinct challenges and opportunities for adaptation within the unique cultural and institutional environment of China. Culturally, many foundational SEL concepts, such as autonomy, personal choice, and the primacy of subjective experience, are rooted in individualistic values (Chong & Lee, 2015). These may conflict with the collectivist orientation prevalent in East Asian societies, where maintaining interpersonal and social harmony is often prioritized over individual expression (Markus & Kitayama, 1991). In such contexts, emotional restraint can be viewed not as maladaptive, but as a culturally valued form of self-regulation (S. X. Chen et al., 2005). Strong emotional displays, by contrast, may be perceived as immaturity or a lack of self-control (Sue & Sue, 2013). This cultural divergence necessitates careful localization of SEL curricula, which often encourage emotional expression and autonomous decision-making (Chong & Lee, 2015).

At the institutional and socio-ecological level, structural inequalities and intense competition for limited high-quality educational resources have led to a strong emphasis on academic achievement, often at the expense of non-cognitive skills like SEL (Dello-Iacovo, 2009; Kipnis, 2011). This performance-driven environment requires SEL programs to demonstrate their practical value within a system largely oriented toward test outcomes. Moreover, the nature of China’s education system, with its focus on academic performance metrics, offers little institutional incentive for adopting non-exam subjects such as SEL. Successful implementation thus demands not only organizational support but also significant shifts in teaching practices and teacher professional development (Fu et al., 2025; Zhong, 2021).

Compared to well-established SEL practices in Western contexts, exemplified by evidence-based programs such as PATHS, Second Step, and Strong Kids (Lawson et al., 2019), school-based SEL in China remains in its early stages. Although notable initiatives exist in China, such as the UNICEF-Ministry of Education collaborative “Social and Emotional Learning and School Management Improvement Project” (Mao et al., 2018), the field still lacks systematic, scalable intervention models and robust effectiveness evidence. Nevertheless, emerging empirical studies in China report positive outcomes associated with SEL interventions. For example, Shi and Cheung (2024b) found that a 12-week SEL curriculum significantly improved fifth graders’ social-emotional competencies. Similarly, Li and Hesketh (2024) observed reduced psychosocial difficulties among rural children both post-intervention and at a 5-month follow-up, despite some attenuation of effects over time. Furthermore, Fu et al. (2024), in a large-scale rural study, documented meaningful gains in overall SEL competencies (ES = 0.213), with left-behind children and boys benefiting more. These findings underscore the applicability and promise of SEL within diverse Chinese school settings.

### 1.3. Current Study

This study employs a meta-analysis to systematically evaluate PreK-12 school-based SEL interventions implemented in the Chinese context, drawing on empirical literature published in both English and Chinese. It aims to address the following research questions:

(1) What is the overall effect of school-based SEL interventions on PreK-12 students in China?
(2) What characteristics moderate the effectiveness of these interventions?

By assessing the impact of SEL within a distinctive setting marked by collectivist cultural values and a highly competitive educational environment, this research seeks to generate robust meta-analytic evidence. The findings are anticipated to inform the design and implementation of holistic education programs within Chinese context, thereby advancing evidence-based practices for localized SEL initiatives and contributing a meaningful Chinese perspective to the global SEL knowledge base.

## 2. Methods

Consistent with the PRISMA statement (Page et al., 2021), we performed this systematic review and meta-analysis. The PRISMA 2020 checklist is available in Supplemental Table S1. To enhance transparency, we note that this review was not preregistered.

### 2.1. Literature Search Strategies

A systematic literature search was conducted to identify relevant studies on school-based SEL interventions. The search was inclusive of publications in both Chinese and English but exclusive of those in other languages. The search aimed to be inclusive of various studies and encompassed all records available up to 30 September 2025. The search strategy involved three complementary approaches. First, systematic electronic searches were performed across four major databases: the China National Knowledge Infrastructure (CNKI), Web of Science, ProQuest, and ERIC. The search strategy incorporated keywords related to (a) SEL constructs (e.g., social-emotional learning, emotional intelligence, non-cognitive skills), (b) intervention (e.g., curriculum, program, trial), (c) setting and population (e.g., school, student, pupil), and (d) geographical context (China OR Chinese). Second, we manually examined the reference lists of prior relevant meta-analyses and systematic reviews to identify additional studies. Third, gray literature and additional Chinese-language reports were identified through web search engines (e.g., Baidu). A complete list of the search terms used in the current study is provided in Supplemental Table S2.

### 2.2. Criteria for Inclusion and Exclusion

The following criteria were used to determine study eligibility for inclusion:

1. The intervention must be a school-based SEL program directed at the general student population. Studies targeting only clinical or special needs subgroups were excluded.
2. It must explicitly aim to evaluate the impact of an SEL intervention on student outcomes.
3. It must employ a controlled research design, featuring both an intervention group and a control group.
4. To ensure baseline equivalence, the pretest difference between the intervention and control groups must not exceed 0.25 standard deviations (What Works Clearinghouse, 2022). Consistent with previous meta-analytic reviews (e.g., Shi & Cheung, 2024a), group differences exceeding this benchmark may confound effect estimates.
5. The total sample size must be at least 60 (with a minimum of 30 participants in both the intervention and control groups). This cutoff was applied because studies with small samples are prone to overestimating effect sizes and is consistent with previous reviews (Shi & Cheung, 2024a).
6. The intervention must have a duration of at least six weeks.
7. The study must report sufficient quantitative data for the calculation of effect sizes.

**Figure 1:**
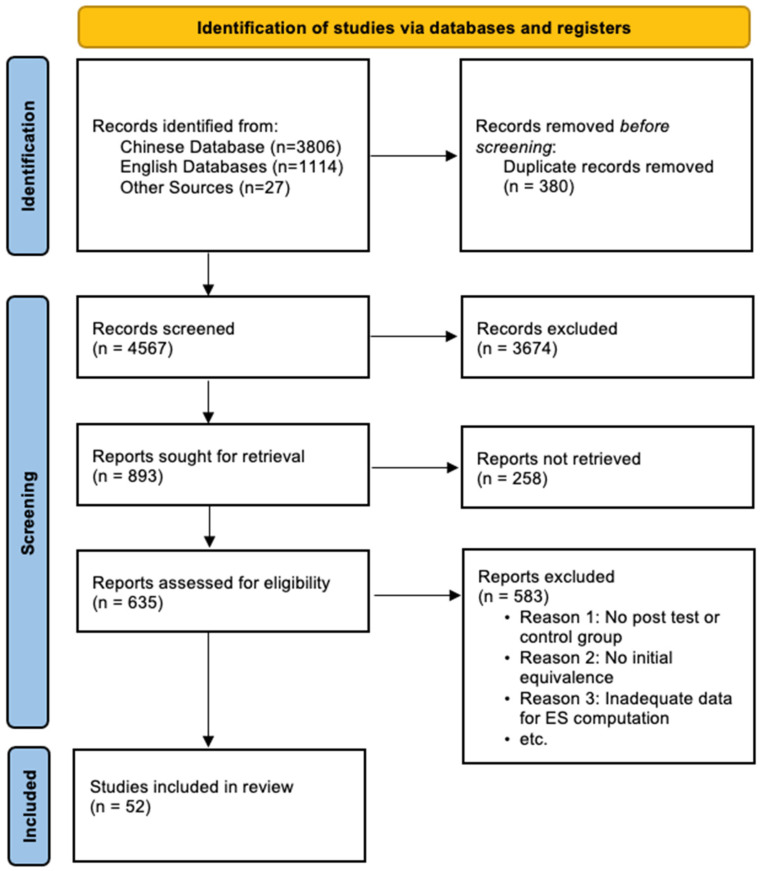
PRISMA Flow Chart of Searching Procedures.

The initial literature search yielded a total of 4947 records. Two researchers independently screened these records based on the above criteria, first by reviewing titles and abstracts, and subsequently by assessing the full texts. Any discrepancies between the researchers were resolved through discussion. A final sample of 52 studies met all inclusion criteria, and their full references are provided in Table S3. The study selection process is detailed in the PRISMA flow diagram (Figure 1).

### 2.3. Risk of Bias Assessment

The risk of bias for each included study was formally evaluated using the Risk Of Bias In Non-randomized Studies—of Interventions (ROBINS-I) tool (Sterne et al., 2016). Two authors independently assessed seven domains of bias: confounding, selection of participants, classification of interventions, deviations from intended interventions, missing data, measurement of outcomes, and selection of the reported result. Each domain, as well as the overall risk, was judged as low, moderate, serious, or critical risk of bias. Discrepancies were resolved through consensus.

### 2.4. Data Extraction and Coding

A standardized coding protocol was developed to extract data from each eligible study, capturing information across four domains: (a) methodological characteristics, (b) intervention features, (c) recipient characteristics, and (d) intervention outcomes, which was similar to previous research (Cipriano et al., 2023; Sklad et al., 2012). Two researchers independently coded all studies. Any discrepancies in coding were resolved through discussion to ensure consensus. Inter-rater agreement between two researchers was assessed using Cohen’s Kappa (k = 0.86).

**Methodological Characteristics:** The following methodological variables were coded. (1) Publication type: Studies were classified as either a published article or a thesis. (2) Research design: Studies were categorized as randomized controlled trial (RCT) or quasi-experimental designs (QED). (3) Sample size: Following previous literature (Cheung & Slavin, 2016; Shi et al., 2025), studies were dichotomized into small (n < 250) and large (n ≥ 250) samples. (4) Duration: Intervention length (in weeks) was coded as a continuous variable. (5) Source of outcome data: Each outcome measure was coded based on its source as student, other (e.g., teacher, parent, observer, school records, or task-based assessment), or mixed.

**Intervention Features:** Key aspects of the interventions were coded as follows. (1) Intervention approach: Based on the level of school participation and integration (Shi et al., 2025), interventions were categorized as either single-component (e.g., involving only a stand-alone curriculum or group counseling) or comprehensive (e.g., involving at least two coordinated components such as student instruction, teacher training, family engagement, or school-wide climate initiatives). This categorization corresponds directly to the predominant school-based SEL implementation models (Du & Mao, 2019), with the single-component approach reflecting a curriculum model and the comprehensive approach reflecting a systemic reform model. (2) Intervention origin: To operationalize cultural adaptation, interventions were classified by their developmental roots into two categories: adapted international programs (e.g., those referencing the CASEL framework or Western SEL curricula) versus indigenous programs (e.g., homegrown interventions informed by local frameworks and embedded in routine school practices). This distinction tests whether grassroots approaches aligned with local cultural norms differ in effectiveness from imported programs. (3) Dosage: Defined as the total time of SEL instruction per week (in minutes) and coded as a continuous variable. (4) Implementer: The primary intervention agent was coded as teacher, researcher, other, or was marked unknown.

**Recipient Characteristics:** Characteristics of the study samples were captured by the following. (1) Grade level: Studies were coded as targeting preschool (students aged 3–6), elementary (students aged 6–12) or secondary school students (aged 12–18). (2) General area of school: Studies were classified based on the general area of the participating schools as rural, suburban, or urban.

**Intervention outcomes:** In line with the prior literature (Durlak et al., 2011; Sklad et al., 2012; Shi & Cheung, 2024a), the outcomes of school-based SEL interventions were categorized into four domains. (1) Social-emotional skills: This domain encompasses the core competencies defined by CASEL, which include self-awareness, self-management, social awareness, relationship skills, and responsible decision-making. It also includes related skills such as emotional intelligence, self-esteem, self-concept, self-efficacy, self-control, empathy, problem-solving, conflict resolution, social skills, and leadership. (2) Affect and attitudes: This category captures students’ internalizing states and their perceptions of their social and academic environments. It includes measures of emotional distress (e.g., anxiety, depression, stress) and psychological well-being, as well as attitudes toward and bonds with peers, teachers, and the school itself (e.g., school bonding, school climate, peer relationships, and teacher-student relationships). (3) Behaviors: This domain involves observable positive and negative behaviors. It includes prosocial behaviors (e.g., altruism, cooperation) and antisocial or problematic behaviors, such as aggression, bullying, violence, disruptive conduct, and substance abuse. (4) Academic performance: This category includes direct measures of academic achievement, including but not limited to standardized test scores in reading and mathematics, as well as broader assessments of academic competence or grade point average. Notably, some constructs assessed in the included studies (e.g., peer problems) encompass multidimensional features that could reasonably be classified into more than one outcome domain (e.g., blending social-emotional skills with behavioral components). To ensure consistency and comparability with major prior SEL meta-analyses (Durlak et al., 2011; Sklad et al., 2012), our coding adhered to the categorical framework adopted in those reviews.

To ensure consistent interpretation, all outcome measures were coded such that higher values indicate more desirable results. Consequently, for negative outcomes (e.g., depression, aggression), the scale was reversed prior to analysis. A positive effect size thus indicates that the SEL intervention was successful in improving positive outcomes (e.g., increasing social skills) or in reducing negative outcomes (e.g., decreasing anxiety). Conversely, a negative effect size suggests that the intervention was associated with a decrease in positive outcomes or an increase in negative ones.

### 2.5. Effect Size Calculations and Statistical Analysis

Effect size is a standardized metric that quantifies the magnitude of an intervention’s impact by expressing it in common standard deviation units, which facilitates direct comparison across studies and outcomes (Kraft, 2020). In this study, we quantify effect sizes using the standardized mean difference (Cohen’s d). It was calculated as the difference between the means of the intervention and control groups, divided by the pooled standard deviation (Cohen, 1988). The formula is presented below:

$$d=\frac{\bar{X_{1}}-\bar{X_{2}}}{S_{pooled}}$$

When means and standard deviations were not directly reported in the primary studies, other available statistical indices (such as t-values, F-statistics, or correlation coefficients) were converted to Cohen’s d following established procedures (Borenstein et al., 2009). For each outcome in each included study, we calculated the final effect size as the difference between post-test and pretest Cohen’s d values, thereby adjusting for any baseline differences between intervention and control groups. All Cohen’s d values were converted to Hedges’ g to compensate for the impacts of the sample size (Hedges, 1981), as the included studies varied substantially in the sample size. All statistical analyses, including the calculation of overall effect sizes, tests of heterogeneity, assessments of publication bias, and subgroup analyses, were performed using Comprehensive Meta-Analysis (CMA) software version 3.

## 3. Results

### 3.1. Research Characteristics

The sample consisted of 52 studies involving a total of 13,969 students. Key characteristics of the included investigations are summarized in Table 1. Regarding methodological characteristics, the majority of the studies (82.7%) were unpublished theses. Nearly all (96.2%) of the studies employed quasi-experimental designs. Most (88.5%) of the studies had a small sample size (fewer than 250 students). Across studies, the mean duration was approximately 18.0 weeks (SD = 14.9), with a range from 7.0 weeks to 15 months. Student self-report was the primary source of outcome data (78.8% of the measurements), followed by other informants (21.2%). With respect to intervention features, single-component programs (84.6%) were considerably more common than comprehensive ones (15.4%). Indigenous programs accounted for 65.4% of the sample, compared to 34.6% for adapted international programs. The intervention dosage varies from 20 to 90 min per week. Researchers served as the implementers in half of the studies, while teachers delivered the intervention in only 15.4%. Geographically, most of the studies were conducted in urban schools (90.4%).

As for recipients’ characteristics, more than half the programs (51.9%) targeted secondary school students, 28.8% involved elementary school students, and the remainder included preschool students. Furthermore, intervention content aligned with a developmental spectrum. Programs for younger students often emphasize emotional moderation and interpersonal harmony, whereas adolescent interventions increasingly address identity exploration and coping with academic pressure. In terms of cultural adaptation, we observed a continuum ranging from direct adaptation of Western curricula, to infusion into existing moral education frameworks, to fully indigenous interventions designed for specific local contexts (e.g., supporting left-behind children).

### 3.2. Publication Bias

**Figure 2:**
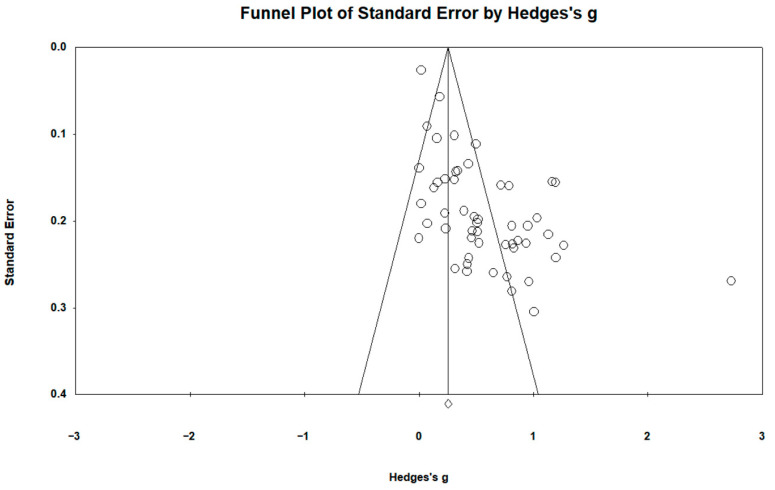
Funnel Plot of Standard Error by Hedge’s g.

Publication bias, the phenomenon whereby studies with statistically significant and positive results are more likely to be published, was assessed using multiple methods. First, the funnel plot (Figure 2) showed an asymmetric distribution of effect sizes, with a greater concentration on the right side of the overall mean effect. Second, Egger’s linear regression test indicated statistically significant funnel plot asymmetry (intercept = 3.37, *p* < 0.001), indicating the existence of publication bias. The positive intercept suggests that smaller studies may have reported larger effect sizes, implying that the pooled effect could be overestimated due to publication bias. Third, the Classic fail-safe N test indicated that 3482 missing studies would be required to render the overall effect statistically non-significant. Similarly, Orwin’s fail-safe N test demonstrated that 1276 missing studies would be needed to reduce the combined effect to a trivial level (set at 0.01). However, because traditional metrics like Egger’s test and fail-safe N are highly sensitive to substantial between-study heterogeneity, we incorporated Duval and Tweedie’s trim-and-fill method as a robust complementary diagnostic. Using a random-effects model, the trim-and-fill analysis indicated that exactly zero missing studies needed to be imputed on the left side of the funnel plot to achieve symmetry. This pivotal finding demonstrates that the observed funnel plot asymmetry is highly unlikely to be an artifact of publication bias (i.e., missing null findings). Instead, it is primarily driven by the substantial internal heterogeneity and structural small-study effects identified in our moderator analyses. Nonetheless, since publication bias remains an inherent and uncontrollable threat in meta-analytic research, the findings of this review should be interpreted with appropriate caution.

### 3.3. Risk of Bias in Included Studies

The results of the risk of bias assessment are presented in the traffic light plot (see Supplementary Figure S1). Overall, the majority of the included studies were judged to have a “serious” overall risk of bias. This rating was predominantly driven by Domain 1 (bias due to confounding), which is an inherent limitation of the quasi-experimental designs heavily utilized in Chinese school-based SEL research. Conversely, most studies demonstrated low risk of bias in the selection of participants (Domain 2) and classification of interventions (Domain 3), reflecting prospective, whole-group inclusion designs. Furthermore, lack of outcome assessor blinding (Domain 6) and absence of study pre-registration (Domain 7) universally resulted in moderate risk across these respective domains.

### 3.4. Overall Effects

A random-effects model was employed for the meta-analysis, based on the assumption that the true effect sizes vary across the included studies (Borenstein et al., 2009). Table 2 showed that the overall effect size of Chinese school-based SEL programs on students was 0.56 (CI = [0.45–0.68], *p* < 0.001). The effect sizes of the studies were strongly heterogeneous. The Q value reached 0.01 significance level, indicating that there was significant heterogeneity among studies (Q = 434.25, df = 51, *p* < 0.001). I^2^ value was larger than 75%, which explained a high degree of heterogeneity (I^2^ = 88.26%). These results suggested the possibility of moderators and laid the foundation for the subsequent analysis of moderation effects.

An examination of the effects across different outcome domains showed that SEL programs had the strongest impact on social-emotional skills (ES = 0.63, k = 42). Significant positive effects were also observed on affect and attitudes (ES = 0.24, k = 13), behaviors (ES = 0.31, k = 15), and academic performance (ES = 0.34, k = 4), indicating that SEL was effective.

The effect sizes for individual studies are presented in the forest plot shown in Figure S2. To assess the robustness of the overall findings, a “one study removed” operation was performed (Borenstein et al., 2009). The results confirmed that the pooled effect size remained stable, ranging from 0.53 to 0.58, and all values remained within the original 95% confidence interval. This indicates that the overall result was not disproportionately influenced by any single study.

### 3.5. Moderator Analysis

To investigate potential sources of the significant heterogeneity observed among the included studies, we examined a set of moderators pertaining to methodological characteristics, intervention features, and recipient characteristics. Notably, two potential moderators, research design and general school area, had a highly uneven distribution of studies across their respective categories. Specifically, there were only 2 RCTs compared to 50 quasi-experimental designs, and for school area, 47 studies were in urban settings, 1 in suburban, and 4 in rural areas. Given that effect size estimates become highly unstable and unreliable when subgroups contain very few studies, these two moderators were excluded from the formal quantitative synthesis to avoid drawing biased or spurious conclusions from such comparisons (Borenstein et al., 2009). Consequently, the following eight moderators were tested: publication type, sample size, duration, source of outcome data, intervention approach, dosage, implementer, and grade level. The results are shown in Table 3.

#### 3.5.1. Publication Type

Given the potential for publication bias indicated in Section 3.2, we examined whether publication type moderated the overall effect. The results showed that the effect size for published articles (ES = 0.57, k = 9) was nearly identical to unpublished theses (ES = 0.56, k = 43), and the difference was not statistically significant (*p* > 0.05). This suggests that publication type did not moderate the intervention effectiveness.

#### 3.5.2. Sample Size

Sample size is a recognized methodological factor that can influence effect size estimates, with larger samples typically yielding more conservative estimates (Cheung & Slavin, 2016; Shi & Cheung, 2024a). In this review, the effect size of small studies (ES = 0.62, k = 46) was significantly larger (*p* < 0.001) than that for studies with large samples (ES = 0.19, k = 6). This substantial difference indicates that the overall effect may be inflated by the predominance of smaller-scale studies.

#### 3.5.3. Source of Outcome Data

Studies were categorized based on the informant of the outcome data: student report, or other report. No statistically significant differences were found between these subgroups (*p* > 0.05). The effect size for studies relying on student reports (ES = 0.55, k = 41) was slightly lower than for those using other informants (ES = 0.62, k = 11), a pattern that aligns with previous research (Shi et al., 2025).

#### 3.5.4. Intervention Approach

Interventions were classified as either single-component or comprehensive based on the level of school participation and integration. The analysis found no significant difference (*p* > 0.05) in effect sizes between single-component (ES = 0.57, k = 44) and comprehensive approaches (ES = 0.53, k = 8). This result suggests that, in this particular context, a more complex, multi-faceted intervention strategy did not yield a discernible advantage over a focused, single-component program.

#### 3.5.5. Intervention Origin

The moderator analysis revealed no statistically significant difference in effectiveness between the two categories (*p* > 0.05). Although indigenous programs yielded a slightly larger overall effect size (ES = 0.60, k = 34) compared to adapted international programs (ES = 0.46, k = 18), this variance did not reach statistical significance. This indicates that both origin pathways are comparably effective in promoting student outcomes.

#### 3.5.6. Implementer

The role of the implementer was hypothesized as an important factor affecting the effect sizes (Wigelsworth et al., 2016). In this review, the subgroup analysis revealed that implementer type was significantly associated with SEL effectiveness (*p* < 0.05). Interventions delivered by researchers yielded the highest effect size (ES = 0.60, k = 26), followed by those delivered by others (ES = 0.48, k = 4), and then by teachers (ES = 0.25, k = 8). A notable subset of studies that did not specify the implementer also showed a high effect size (ES = 0.70, k = 14). The comparatively lower effect sizes for teacher-led interventions are consistent with the previous findings (Durlak et al., 2011).

#### 3.5.7. Grade Level

Consistent with prior research (Corcoran et al., 2018), grade level was found to be a significant moderator of SEL intervention effects (*p* < 0.01). The largest effects were observed in preschool (ES = 0.71, k = 10), followed by secondary school (ES = 0.65, k = 27), with the smallest effects in elementary school (ES = 0.33, k = 15). This non-linear pattern across developmental stages underscores the importance of tailoring interventions to the specific needs and receptivity of different age groups.

### 3.6. Meta-Regression and Confounding Analysis

To simultaneously examine the effects of multiple moderators, treat continuous variables appropriately, and address potential confounding relationships (e.g., sample size, implementer, and grade level), we conducted a stepwise random-effects meta-regression analysis. The key models (Models 1–5) are presented in Table 4.

The results identified Model 2 (incorporating sample size and implementer) as the most parsimonious and robust model, explaining approximately 42% of the between-study heterogeneity (R^2^ analog = 0.42). In this model, sample size remained a significant negative predictor of effect sizes. Furthermore, introducing continuous variables for dosage (Model 3) and duration (Model 4) neither yielded significant coefficients nor improved the model’s explanatory power. Further exploratory models combining these non-significant continuous variables with other moderators consistently reduced the R^2^ analog and yielded no significant predictors, confirming that intervention duration and dosage were not primary drivers of effectiveness in this dataset.

Crucially, when grade level was introduced into the optimal model alongside sample size and implementer (Model 5), the overall model fit decreased (R^2^ analog = 0.33), and the previously significant effect of sample size disappeared. This attenuation indicates substantial structural confounding (multicollinearity) among these variables. To unpack this confounding, a closer examination of study characteristics across grade levels was conducted (see Supplemental Table S4). This descriptive analysis revealed a strong structural overlap between grade level and sample size. Specifically, preschool (100%) and secondary school (92.6%) studies were predominantly small scale, making them highly prone to effect inflation. In contrast, elementary school studies included a notably larger proportion of large-scale trials (26.7%). Furthermore, implementation characteristics also differed, with researcher involvement being substantially higher in preschool and elementary settings (60% each) than in secondary schools (40.7%). Consequently, these findings suggest that any observed differences across grade levels are heavily intertwined with underlying variations in study scale and implementation fidelity, rather than representing purely developmental differences in SEL effectiveness.

## 4. Discussion

### 4.1. The Overall Effect of School-Based SEL Interventions

This study represents the first meta-analysis of universal, school-based SEL interventions in China’s PreK-12 context. A total of 52 eligible studies involving 13,969 students were included. The results indicate that school-based SEL interventions in China yield a significant, positive overall effect (ES = 0.56). With respect to specific outcome domains, SEL programs showed the strongest effect on the development of social-emotional skills (ES = 0.63), followed by academic performance (ES = 0.34), behavioral outcomes (ES = 0.31), and affect and attitudes (ES = 0.24). According to Cohen’s (1988) conventional benchmarks for effect sizes, where values of 0.2, 0.5, and 0.8 represent small, medium, and large effects, the overall effect observed in this review falls into the medium range. This finding is highly consistent with earlier meta-analyses conducted in Western contexts, such as those by Durlak et al. (2011; ES = 0.57) and Wigelsworth et al. (2016; ES = 0.53), suggesting that despite China’s distinct collectivist culture and competitive academic environment, SEL interventions are effective in improving student outcomes.

The relatively large overall effect size may be partially attributable to the predominance of urban, small-sample, quasi-experimental, and researcher-led studies among the included studies. This overall estimate (ES = 0.56) should be interpreted primarily as an outcome derived largely from controlled, optimal conditions rather than scale-up practice. Notably, our analysis revealed that small-scale, researcher-led trials yielded a large effect (ES = 0.62), whereas larger-scale studies produced a significantly smaller effect (ES = 0.19). This discrepancy indicates a potential scalability drop-off. The larger-scale estimate (ES = 0.19) aligns closely with recent rigorous international reviews (e.g., Cipriano et al., 2023, ES = 0.22; Shi & Cheung, 2024a, ES = 0.17) and likely represents a more realistic benchmark for system-wide implementation. Regarding potential mechanisms, as interventions expand from tightly controlled trials to real-world implementation, effect sizes often attenuate due to reduced novelty, lower implementation fidelity, and weaker treatment–control contrasts (Wigelsworth et al., 2016). This pattern is consistent with the phased intervention development framework (Campbell et al., 2000), where early-stage trials yield stronger effects under researcher-supported conditions, while later scaling stages face resource limitations and contextual variability that may dilute outcomes (Durlak & DuPre, 2008). Thus, while the current evidence confirms that SEL has strong potential in China, estimates of its impact under real-world conditions warrant more conservative expectations.

### 4.2. Moderators of Intervention Effectiveness

The current review examined nine potential moderators of SEL program effectiveness, including publication type, sample size, duration, source of outcome, intervention approach, intervention origin, dosage, implementer, and grade level. Univariate subgroup analysis showed that sample size, implementer, and grade level were statistically significant moderators, whereas other variables did not reach significance. With respect to methodological characteristics, sample size emerged as a predominant moderator. Small studies (n < 250) yielded an effect size 0.43 higher than that of large studies. This aligns with prior meta-analytic findings that smaller studies often report inflated effects (Corcoran et al., 2018), possibly due to lower sampling variability and more controlled implementation contexts (Cheung & Slavin, 2016). While small-scale trials establish initial efficacy, larger samples provide a more realistic estimate of effectiveness for generalization (Slavin & Smith, 2009).

Regarding intervention features, the implementer played a critical role. Programs implemented by researchers yielded the largest effect sizes, a result that aligns with earlier findings (Wigelsworth et al., 2016). Eisner (2009) attributed such developer-implementer effects to either systematic bias (the cynical view) or enhanced implementation fidelity (the high-fidelity view). Aligning with recent findings by Shi and Cheung (2024a), intervention duration did not significantly moderate outcomes in our analysis. However, contrary to their report, dosage failed to reach significance in our sample. This specific inconsistency regarding dosage may be largely attributable to variations in how dosage thresholds and intervention intensity are operationalized across primary studies. This suggests that within the Chinese context, “how long” an intervention lasts may be less critical than “how well” it is implemented. It is also possible that short-term interventions in this review were disproportionately researcher-led and intensive, thereby masking any potential benefits of longer duration through high implementation fidelity. Furthermore, regarding intervention origin, our analysis revealed no statistically significant difference between adapted international frameworks (ES = 0.46) and indigenous programs (ES = 0.60). From a cultural adaptation perspective, this non-significant divergence is a highly encouraging finding. This suggests homegrown SEL interventions, embedded in local frameworks and practices, are comparably effective to localized Western blueprints. Thus, SEL effectiveness in China may depend less on importing structured models than on alignment with the immediate educational context.

As for the recipient characteristic, grade level emerged as a significant moderator, with the largest impacts observed in preschool (ES = 0.71) and secondary school (ES = 0.65), compared to elementary school (ES = 0.33). This pattern aligns with the trend reported by Shi et al. (2025), although the differences between grade levels in that study were not statistically significant. While theoretical perspectives on developmental plasticity (Cunha & Heckman, 2007) and adolescent social sensitivity (Blakemore & Mills, 2014) might explain these peaks, these developmental interpretations must be viewed with caution (Yeager et al., 2018). As demonstrated by the multivariate meta-regression and confounding analyses, these grade-level differences are largely washed out when accounting for sample size and implementer type. Because small-scale, researcher-led trials are disproportionately concentrated in preschools and secondary schools, their respective effect sizes are artificially inflated. Therefore, the observed variation across grade levels is primarily an artifact of methodological confounding rather than a reflection of pure developmental receptivity. Consequently, any conclusions regarding the “optimal age” for SEL in China remain tentative.

### 4.3. The Current Evidence Base for School-Based SEL in China

When interpreting the findings of this meta-analysis, it is essential to consider the nature of the underlying evidence, as its characteristics directly shape the validity and generalizability of the pooled results. The current literature on school-based SEL in China is primarily composed of small-scale, researcher-led studies conducted in urban settings, with a predominance of quasi-experimental designs and published in theses. This composition aligns with an early, efficacy-focused stage of intervention research (Campbell et al., 2000).

Two principal validity considerations emerge from the evidence base. First, the external validity of the overall effect (ES = 0.56) is constrained because the findings are derived largely from conditions that differ from routine school practice. The predominance of quasi-experimental designs and the frequent use of waitlist or business-as-usual control conditions may inflate effect sizes due to a pronounced treatment contrast (Kraft, 2020). This means the estimate, to some extent, reflects efficacy under supported, researcher-led circumstances rather than effectiveness under routine, teacher-led implementation. Consequently, its generalizability to typical public schools, especially in under-resourced or rural settings, remains uncertain (Fixsen et al., 2009; Durlak & DuPre, 2008). Second, concerns regarding ecological validity by age arise from the over-representation of secondary school students in the literature and the relative scarcity of studies in preschool and elementary grades. This uneven distribution limits balanced conclusions about SEL effectiveness across the full PreK–12 continuum and may obscure developmentally specific mechanisms and needs.

The geographic and demographic concentration of the existing evidence base raises important equity concerns. Studies have been conducted overwhelmingly in urban schools, meaning that the direct applicability of findings to rural, migrant, or left-behind children is inferred rather than empirically demonstrated. We acknowledge this urban-rural evidence gap as a major limitation affecting the study’s conclusions, as it restricts the generalizability of our findings primarily to resource-rich urban contexts, leaving the effectiveness of SEL in rural settings largely unverified. This uneven representation creates a form of evidence inequality: those students most in need of tailored SEL supports are precisely those least visible in the evaluation literature (Heckman & Mosso, 2014). Therefore, recommendations for extending SEL to underserved communities should be explicitly framed as forward-looking, justice-oriented imperatives for both research and policy, aimed at closing this representational gap and advancing educational equity (Espinoza, 2007).

### 4.4. Implications and Limitations

Several limitations of the current review should be acknowledged. First, the absence of a preregistered protocol may raise concerns regarding analytical flexibility. We mitigated this risk by adhering to established reporting guidelines (e.g., PRISMA) and systematically documenting all coding decisions and inclusion criteria. In addition, by restricting our search to studies published in English and Chinese, we may have introduced a language bias, potentially excluding relevant findings published in other languages or regional journals. Future research would benefit from preregistered protocols and multilingual searches to enhance both transparency and inclusivity. Second, the relatively small number of included studies limited the ability to investigate a wider array of potential moderators and precluded meta-regression analyses of their combined effects. Additionally, the reliance on median splits to categorize continuous variables (duration and dosage), while necessary to ensure balanced subgroups, may have obscured more nuanced dose–response relationships or specific threshold effects. Future research incorporating a larger body of primary research, along with more detailed methodological, intervention, and participant characteristics, is needed to enhance the robustness and comprehensiveness of the evidence base. Third, there might be other potential moderators that influence SEL intervention effectiveness, such as specific instructional strategies, implementation quality, or school-level policy support. Future studies should explore these factors to develop a more contextualized and conditional understanding of how SEL programs succeed within Chinese educational settings. Last but not least, reflecting the strong emphasis on academic achievement within China’s education system, it is noteworthy that only 4 of the 52 studies included in this meta-analysis examined academic performance outcomes. This gap underscores the need for more research to explicitly investigate how SEL interventions affect Chinese students’ academic achievement.

This meta-analysis provides the first systematic evidence that school-based SEL interventions implemented in the Chinese PreK-12 context exert significant, positive effects on student development. However, the field in China remains at a nascent stage. Most included studies are characterized by quasi-experimental designs, researcher-led implementation, small-scale delivery, and short durations. Moving forward, three strategic directions are recommended to advance the field. First, intervention programs require refinement in both content and methodology. Regarding content, it is crucial to ensure developmental alignment, for instance, by tailoring instruction in emotion concepts and peer dynamics to specific age groups. Concurrently, SEL content must move beyond surface structure adaptations to deep structure adaptations that resonate with Chinese cultural values. Our empirical finding that indigenous programs perform just as well as adapted international programs strongly underscores the viability of grassroots educational innovation. It implies that rather than relying solely on the structural importation of Western curricula, Chinese educators can successfully cultivate SEL programs that inherently align with local cultural norms, thereby avoiding potential cross-cultural friction. Our review highlights the promise of the “infusion approach,” where SEL is embedded into statutory Moral Education or class management systems. By aligning with these indigenous frameworks, interventions can bypass the “crowded curriculum” barrier better than imported standalone programs. Methodologically, employing more rigorous designs, such as randomized controlled trials implemented across real-world settings, would strengthen this evidence base.

Second, greater attention must be paid to implementation processes and systemic integration. As interventions transition from controlled settings to real-world schools, which function as bureaucratic systems, their effectiveness becomes highly dependent on how they are integrated into existing structures. In a high-stakes testing environment, framing SEL solely as mental health often relegates it to a peripheral status. Instead, future efforts should theoretically explicate how SEL mechanisms, specifically self-regulation and grit, serve as non-cognitive drivers of academic achievement. This aligns with our finding of a robust academic effect (ES = 0.34). By reframing SEL as a scaffold for mastering China’s rigorous curriculum, practitioners can secure the leadership support and teacher readiness required for sustainable implementation (Durlak & DuPre, 2008; Fu et al., 2025).

Third, future research should prioritize inquiry focused on rural and vulnerable student populations. From an educational justice perspective, and in line with China’s social context, more targeted SEL interventions should be designed and evaluated for vulnerable student subgroups, such as rural and left-behind children. Such work would not only address pressing social disparities but also generate evidence on the adaptability of SEL across diverse populations (Heckman, 2006). It is hoped that future research will yield more diverse and robust school-based SEL studies in China, thereby contributing to evidence-based practice locally and offering meaningful comparative insights for the global SEL research. Finally, to contribute to the global knowledge base on SEL, future research should include studies from diverse cultural contexts and test cultural factors as moderators. This would help to clarify the role of China as a distinct cultural setting within cross-cultural research on SEL.

## 5. Conclusions

This meta-analysis provides the first systematic synthesis of universal school-based SEL interventions within the PreK-12 context in China. The findings demonstrate that SEL programs implemented in Chinese schools yield a significant, medium overall effect (ES = 0.56) across multiple student outcomes, most notably in social-emotional skills (ES = 0.63), followed by academic performance (ES = 0.34), behaviors (ES = 0.31), and affect and attitudes (ES = 0.24). Importantly, these positive effects are consistent with those observed in Western settings, underscoring the cross-cultural relevance and applicability of SEL frameworks. Key factors such as intervention dosage, implementer involvement, and student grade level were identified as meaningful moderators of program success, offering practical insights for future intervention design and implementation. Despite the promising evidence, the field in China remains in an early stage, characterized by small-scale, researcher-led quasi-experiments. Future research should prioritize culturally grounded program development, rigorous methodological designs, and systematic examination of implementation processes as SEL scales in China, thereby advancing evidence-based practice within the country and enriching the global understanding of SEL across diverse educational contexts.

## Figures and Tables

**Table 1:** Descriptive Characteristics of 52 School-Based SEL Interventions.

General Study Features	N	%
**Methodological characteristics**		
Publication type		
Published article	9	17.3
Thesis	43	82.7
Research design		
RCT	2	3.8
QED	50	96.2
Sample size		
Small (<250)	46	88.5
Large (≥250)	6	11.5
Source of outcome data		
Student	41	78.8
Other	11	21.2
**Intervention features**		
Intervention approach		
Single component	44	84.6
Comprehensive	8	15.4
Intervention origin		
Adapted international	18	34.6
Indigenous	34	65.4
Implementer		
Teacher	8	15.4
Researcher	26	50.0
Other	4	7.7
Unknown	14	26.9
**Recipients’ characteristics**		
Grade level		
Preschool	10	19.2
Elementary	15	28.8
Secondary	27	51.9
General area of school		
Rural	4	7.7
Suburban	1	1.9
Urban	47	90.4

**Table 2:** The Mean Effects and Heterogeneity Tests.

Outcome	Effect Size and 95% Confidence Interval					Test of Heterogeneity			
	Number of Studies	Point Estimate	Standard Error	Lower Limit	Upper Limit	Q-Value	*df* (Q)	*p*-Value	I-Squared
Overall ES	52	0.56	0.06	0.45	0.68	434.25	51	0.000	88.26
Social-emotional skills	42	0.63	0.07	0.50	0.76	264.11	41	0.000	84.48
Affect and attitudes	13	0.24	0.06	0.12	0.37	36.14	12	0.000	66.80
Behaviors	15	0.31	0.08	0.15	0.47	92.69	14	0.000	84.90
Academic performance	4	0.34	0.08	0.19	0.49	3.03	3	0.388	0.84

**Table 3:** The results of moderator analysis.

Study Features	Effect Size and 95% Confidence Interval					Test of Heterogeneity		
	Number of Studies	Point Estimate	Standard Error	Lower Limit	Upper Limit	Q-Value	*df* (Q)	*p*-Value
**By publication type**								
Published article	9	0.57	0.14	0.29	0.85			
Thesis	43	0.56	0.05	0.45	0.66			
Total between	52					0.01	1	0.94
**By sample size**								
Small (<250)	46	0.62	0.07	0.50	0.75			
Large (≥250)	6	0.19	0.07	0.06	0.32			
Total between	52					21.26	1	0.000
**By source of outcome data**								
Student	41	0.55	0.06	0.43	0.67			
Other	11	0.62	0.18	0.27	0.97			
Total between	52					0.13	1	0.717
**By intervention approach**								
Single component	44	0.57	0.07	0.45	0.70			
Comprehensive	8	0.53	0.14	0.26	0.81			
Total between	52					0.07	1	0.788
**By intervention origin**								
Adapted international	18	0.49	0.10	0.29	0.68			
Indigenous	34	0.59	0.05	0.49	0.70			
Total between	52					0.90	1	0.343
**By implementer**								
Teacher	8	0.25	0.08	0.09	0.41			
Researcher	26	0.60	0.08	0.45	0.75			
Other	4	0.48	0.21	0.07	0.90			
Unknown	14	0.70	0.15	0.41	0.99			
Total between	52					12.33	3	0.006
**By grade level**								
Preschool	10	0.71	0.15	0.42	1.00			
Elementary	15	0.33	0.06	0.22	0.44			
Secondary	27	0.65	0.10	0.45	0.84			
Total between	52					11.46	2	0.003

**Table 4:** Results of Meta-regression.

Random Effects	Model 1	Model 2	Model 3	Model 4	Model 5
Intercept	0.201 (0.125)	0.274 (0.141)	0.280 (0.222)	0.308 (0.189)	0.226 (0.153)
Sample size (Small)	0.413 ** (0.136)	0.351 * (0.137)	0.351 * (0.143)	0.334 * (0.163)	0.244 (0.156)
Implementer (not researcher)		−0.166 (0.121)	−0.164 (0.123)	−0.153 (0.133)	−0.200 (0.129)
Implementer (NA)		0.080 (0.117)	0.080 (0.120)	0.088 (0.127)	0.064 (0.128)
Dosage			−0.000 (0.003)		
Duration				−0.001 (0.004)	
Grade level (Preschool)					0.286 (0.160)
Grade level (Secondary)					0.200 (0.122)
Q	9.20	13.01	12.38	11.00	15.43
df	1	3	4	4	5
R^2^ analog	0.39	0.42	0.39	0.29	0.33

Standard errors in parentheses; * *p* < 0.05, ** *p* < 0.01.

## Data Availability

The original contributions presented in this study are included in the Supplementary Materials.

## Supplementary Materials

The following supporting information can be downloaded at: https://www.mdpi.com/article/10.3390/bs16030368/s1, Table S1: PRISMA 2020 Checklist; Table S2: Detailed Search Query; Table S3: References of the Included Studies; Table S4: Descriptive Cross-Tabulation of Grade Level and Other Characteristics; Figure S1: Traffic Light Plot of Risk of Bias Assessments for the Included Studies Using the ROBINS-I Tool; Figure S2: Forest Plot of the Effect Sizes.
