# Supplementary material for: Promoting Holistic Student Development Through Universal School-Based Social-Emotional Learning in China: A Meta-Analysis
Source: Behav Sci (Basel). 2026 Mar 5;16(3):368. doi: 10.3390/bs16030368 (PMC13024163; doi:10.3390/bs16030368)
Supplement: Supplementary file 1 [file behavsci-16-00368-s001.zip › behavsci-4141178-supplementary.pdf]

behavioral sciences

MDPI

Supplementary Materials

Table S1. PRISMA 2020 Checklist

 https://doi.org/10.3390/bs16030368

Section and Topic	Item #	Checklist item	Reported on page #
**TITLE**			
Title	1	Identify the report as a systematic review.	1
**ABSTRACT**			
Abstract	2	See the PRISMA 2020 for Abstracts checklist.	1
**INTRODUCTION**			
Rationale	3	Describe the rationale for the review in the context of existing knowledge.	1-4
Objectives	4	Provide an explicit statement of the objective(s) or question(s) the review addresses.	4
**METHODS**			
Eligibility criteria	5	Specify the inclusion and exclusion criteria for the review and how studies were grouped for the syntheses.	4-5
Information sources	6	Specify all databases, registers, websites, organisations, reference lists and other sources searched or consulted to identify studies. Specify the date when each source was last searched or consulted.	4
Search strategy	7	Present the full search strategies for all databases, registers and websites, including any filters and limits used.	4
Selection process	8	Specify the methods used to decide whether a study met the inclusion criteria of the review, including how many reviewers screened each record and each report retrieved, whether they worked independently, and if applicable, details of automation tools used in the process.	5
Data collection process	9	Specify the methods used to collect data from reports, including how many reviewers collected data from each report, whether they worked independently, any processes for obtaining or confirming data from study investigators, and if applicable, details of automation tools used in the process.	6-7
Data items	10a	List and define all outcomes for which data were sought. Specify whether all results that were compatible with each outcome domain in each study were sought (e.g. for all measures, time points, analyses), and if not, the methods used to decide which results to collect.	7
	10b	List and define all other variables for which data were sought (e.g. participant and intervention characteristics, funding sources). Describe any assumptions made about any missing or unclear information.	6-7
Study risk of bias assessment	11	Specify the methods used to assess risk of bias in the included studies, including details of the tool(s) used, how many reviewers assessed each study and whether they worked independently, and if applicable, details of automation tools used in the process.	5

Section and Topic	Item #	Checklist item	Reported on page #
Effect measures	12	Specify for each outcome the effect measure(s) (e.g. risk ratio, mean difference) used in the synthesis or presentation of results.	7
Synthesis methods	13a	Describe the processes used to decide which studies were eligible for each synthesis (e.g. tabulating the study intervention characteristics and comparing against the planned groups for each synthesis (item #5)).	7
	13b	Describe any methods required to prepare the data for presentation or synthesis, such as handling of missing summary statistics, or data conversions.	7
	13c	Describe any methods used to tabulate or visually display results of individual studies and syntheses.	7
	13d	Describe any methods used to synthesize results and provide a rationale for the choice(s). If meta-analysis was performed, describe the model(s), method(s) to identify the presence and extent of statistical heterogeneity, and software package(s) used.	7
	13e	Describe any methods used to explore possible causes of heterogeneity among study results (e.g. subgroup analysis, meta-regression).	7
	13f	Describe any sensitivity analyses conducted to assess robustness of the synthesized results.	10
Reporting bias assessment	14	Describe any methods used to assess risk of bias due to missing results in a synthesis (arising from reporting biases).	7
Certainty assessment	15	Describe any methods used to assess certainty (or confidence) in the body of evidence for an outcome.	7
**RESULTS**			
Study selection	16a	Describe the results of the search and selection process, from the number of records identified in the search to the number of studies included in the review, ideally using a flow diagram.	6
	16b	Cite studies that might appear to meet the inclusion criteria, but which were excluded, and explain why they were excluded.	5
Study characteristics	17	Cite each included study and present its characteristics.	8
Risk of bias in studies	18	Present assessments of risk of bias for each included study.	8-9

Section and Topic	Item #	Checklist item	Reported on page #
Results of individual studies	19	For all outcomes, present, for each study: (a) summary statistics for each group (where appropriate) and (b) an effect estimate and its precision (e.g. confidence/credible interval), ideally using structured tables or plots.	Supplementary Materials S1
Results of syntheses	20a	For each synthesis, briefly summarise the characteristics and risk of bias among contributing studies.	9-10
	20b	Present results of all statistical syntheses conducted. If meta-analysis was done, present for each the summary estimate and its precision (e.g. confidence/credible interval) and measures of statistical heterogeneity. If comparing groups, describe the direction of the effect.	9-12
	20c	Present results of all investigations of possible causes of heterogeneity among study results.	9-12
	20d	Present results of all sensitivity analyses conducted to assess the robustness of the synthesized results.	10
Reporting biases	21	Present assessments of risk of bias due to missing results (arising from reporting biases) for each synthesis assessed.	10
Certainty of evidence	22	Present assessments of certainty (or confidence) in the body of evidence for each outcome assessed.	10
**DISCUSSION**			
Discussion	23a	Provide a general interpretation of the results in the context of other evidence.	12-13
	23b	Discuss any limitations of the evidence included in the review.	13-14
	23c	Discuss any limitations of the review processes used.	14
	23d	Discuss implications of the results for practice, policy, and future research.	14-15
**OTHER INFORMATION**			
Registration and protocol	24a	Provide registration information for the review, including register name and registration number, or state that the review was not registered.	4
	24b	Indicate where the review protocol can be accessed, or state that a protocol was not prepared.	4
	24c	Describe and explain any amendments to information provided at registration or in the protocol.	4
Support	25	Describe sources of financial or non-financial support for the review, and the role of the funders or sponsors in the review.	16
Competing	26	Declare any competing interests of review authors.	16

https://doi.org/10.3390/bs16030368

Section and Topic	Item #	Checklist item	Reported on page #
interests			
Availability of data, code and other materials	27	Report which of the following are publicly available and where they can be found: template data collection forms; data extracted from included studies; data used for all analyses; analytic code; any other materials used in the review.	16

From: Page MJ, McKenzie JE, Bossuyt PM, Boutron I, Hoffmann TC, Mulrow CD, et al. The PRISMA 2020 statement: an updated guideline for reporting systematic reviews. BMJ 2021;372:n71. doi: 10.1136/bmj.n71.

This work is licensed under CC BY 4.0. To view a copy of this license, visit https://creativecommons.org/licenses/by/4.0/

Table S2. Detailed Search Query

This table outlines the complete Boolean search query as applied in the Web of Science database. The strategy was adapted as needed for syntax requirements in other databases (e.g., CNKI, ProQuest, ERIC).

Search Block	Key Concepts	Search Query
**#1**	SEL Constructs	AB=("social emotional learning" OR "SEL" OR "social emotional program" OR "social emotional skill" OR "social emotional competence" OR "emotional intelligence" OR "soft skills" OR "noncognitive skills" OR "social skills" OR "self-awareness" OR "self-management" OR "social-awareness" OR "relationship skills" OR "responsible decision making")
**#2**	Intervention	AB=(intervention OR experiment OR trial OR curriculum OR program OR project)
**#3**	Setting/Population	AB=(school OR kindergarten OR child* OR student OR teacher OR adolescent OR pupil)
**#4**	Geographical Context	AB=(China OR Chinese)
	**Final Combined Query**	**#1 AND #2 AND #3 AND #4**

Note: AB = Abstract.

Table S3. References of the Included Studies

An, L., Vaid, E., Elias, M. J., Li, Q., Wang, M., & Zhao, G. (2021). Promotion of social and emotional learning in a Chinese elementary school. *Social Behavior and Personality: An International Journal*, *49*(10), 1–9. https://doi.org/10.2224/sbp.10625

Cao, L. (2018). *The influence of group counseling on the social behavior of lower-grade primary school students* (in Chinese) [Master's thesis, Wenzhou University].

Chen, L. (2012). *Investigation and intervention research on relational aggression in junior high school students* (in Chinese) [Master's thesis, Shanxi University].

Deng, Y. (2016). *Research on the integration of emotional intelligence education in middle school physical education teaching* (in Chinese) [Master's thesis, Fujian Normal University].

Dong, W. (2020). *The influence of emotional intelligence on social adaptability in high school students and its intervention research* (in Chinese) [Master's thesis, Yunnan Normal University]. https://doi.org/10.27459/d.cnki.gynfc.2020.000843

Fan, Y. (2017). *Experimental research on the cultivation of emotional intelligence in rural junior high school students* (in Chinese) [Master's thesis, Shanxi Normal University].

Fu, L., Zhang, Z., Yang, Y., & Curtis McMillen, J. (2024). Acceptability and preliminary impact of a school-based SEL program for rural children in China: A quasi-experimental study. *Children and Youth Services Review*, *160*, 107579. https://doi.org/10.1016/j.childyouth.2024.107579

Gao, J. (2022). *Research on the development of kindergarten social emotional learning curriculum from the perspective of embodied cognition* (in Chinese) [Master's thesis, Xinyang Normal University]. https://doi.org/10.27435/d.cnki.gxsfc.2022.000407

Gao, Z. (2016). *An empirical study on the impact of social emotional education on externalizing problem behaviors in kindergarten top class children* (in Chinese) [Master's thesis, Shanghai Normal University].

Han, D. (2019). *Intervention research on cultivating emotional intelligence of junior high school students based on thematic mental health activity courses* (in Chinese) [Master's thesis, Yanbian University].

He, H. (2018). *Research on the effectiveness of offering positive mental health education courses in junior high schools* (in Chinese) [Master's thesis, Hebei Normal University].

He, Z. (2025). *Teaching intervention research on peer relationships and self-esteem levels of upper-grade primary school students* (in Chinese) [Master's thesis, Dali University]. https://doi.org/10.27811/d.cnki.gdixy.2025.000413

Hu, K. (2016). *Research on social emotional health intervention for upper-grade primary school students* (in Chinese) [Master's thesis, Hunan Normal University].

Hu, W. (2025). *The relationship between perceived social support and peer relationships in junior high school students: The mediating role of emotional intelligence and intervention research* (in Chinese) [Master's thesis, Chengdu University]. https://doi.org/10.27917/d.cnki.gcxdy.2025.000192

Jia, Y. (2019). *The relationship between emotional intelligence and academic self-efficacy in junior high school students and its intervention research* (in Chinese) [Master's thesis, Inner Mongolia Normal University].

Jiang, Z. (2016). *Practical exploration of implementing emotional intelligence cultivation in high school* (in Chinese) [Master's thesis, Ludong University].

Li, J., & Hesketh, T. (2024). A social emotional learning intervention to reduce psychosocial difficulties among rural children in central China. *Applied Psychology: Health and Well-Being*, *16(*1), 235–253. https://doi.org/10.1111/aphw.12481

Liang, J. (2016). *Intervention research on improving emotional intelligence and psychological resilience of high school students through class group counseling* (in Chinese) [Master's thesis, Shaanxi Normal University].

Lin, H. (2016). *Intervention research on promoting psychological resilience of junior high school students through emotional intelligence-themed mental health education courses* (in Chinese) [Master's thesis, Central China Normal University].

Liu, L. (2017). *Practical research on social and emotional learning curriculum focusing on young children's interpersonal communication* (in Chinese) [Master's thesis, Shanghai Normal University].

Liu, Z. (2024). *Design and implementation of a mental health curriculum to improve emotional regulation self-efficacy in junior high school students* (in Chinese) [Master's thesis, Nanchang University]. https://doi.org/10.27232/d.cnki.gnchu.2024.002525

Lu, Y., Bai, F., & Wen, Y. (2023). Educational experimental research on cultivating emotional intelligence in junior high school students (in Chinese). *Longyan University Journal*, *41*(5), 123–128. https://doi.org/10.16813/j.cnki.cn35-1286/g4.2023.05.020

Ma, Y. (2014). The influence of group counseling on the development of emotional intelligence in middle school students (in Chinese). *Educational Science Research*, (2), 59–62.

Ma, Z. (2018). *Intervention research on improving emotional intelligence of primary school students through class group counseling* (in Chinese) [Master's thesis, Nanjing University of Chinese Medicine].

Niu, L. (2020). *Design and implementation of a curriculum for emotional regulation self-efficacy in junior high school students* (in Chinese) [Master's thesis, Shihezi University]. https://doi.org/10.27332/d.cnki.gshzu.2020.000133

Qi, H. (2019). *Educational experimental research on preventing school weariness in junior high school students through emotional intelligence psychological courses* (in Chinese) [Master's thesis, Zhengzhou University].

Qi, Z. (2011). *Intervention research on emotional literacy of primary school students* (in Chinese) [Master's thesis, Shanghai Normal University].

Shen, H. (2022). *Experimental research on social emotional learning (SEL) curriculum in primary schools* (in Chinese) [Master's thesis, Zhengzhou University]. https://doi.org/10.27466/d.cnki.gzzdu.2022.002909

Shi, J., & Cheung, A. C. K. (2024). The Impacts of a Social Emotional Learning Program on Elementary School Students in China: A Quasi-Experimental Study. *The Asia-Pacific Education Researcher*, *33*(1), 59–69. https://doi.org/10.1007/s40299-022-00707-9

Shi, Y. (2019). *Practical research on improving emotional understanding ability of middle class kindergarten children through collective emotional talk activities* (in Chinese) [Master's thesis, Shanghai Normal University].

Shi, Z. (2017). *Educational experimental research on "social emotional learning" in domestic primary schools* (in Chinese) [Master's thesis, Jiangsu University].

Song, J., Zhang, Y., & Jin, F. (2022). Experimental study on the influence of social emotional learning curriculum on peer interaction ability of 4-6 year old children (in Chinese). *Qiqihar Teachers College Journal*, *5*, 16–20. https://doi.org/10.16322/j.cnki.23-1534/z.2022.05.036

Su, T. (2024). *The relationship between self-acceptance and social anxiety in fourth-grade primary school students and intervention research* (in Chinese) [Master's thesis, Jilin International Studies University]. https://doi.org/10.27833/d.cnki.gjlhw.2024.000499

Sun, H. (2025). *The influence of emotional regulation on anxiety in junior high school students and intervention research* (in Chinese) [Master's thesis, Dali University]. https://doi.org/10.27811/d.cnki.gdixy.2025.000325

Wang, C. (2025). *Practical research on promoting the development of social emotional ability of middle class kindergarten children through picture book teaching* (in Chinese) [Master's thesis, Dali University]. https://doi.org/10.27811/d.cnki.gdixy.2025.000120

Wang, H., Chu, J., Loyalka, P., Xin, T., Shi, Y., Qu, Q., & Yang, C. (2016). Can social–emotional learning reduce school dropout in developing countries? *Journal of Policy Analysis and Management*, *35*(4), 818–847. https://doi.org/10.1002/pam.21915

Wang, H. (2019). *The relationship between emotional intelligence, peer relationships, campus bullying in junior high school students and intervention research* (in Chinese) [Master's thesis, Inner Mongolia Normal University].

Wu, S. (2025). *Practical research on improving social skills of middle class kindergarten children through role-playing games* (in Chinese) [Master's thesis, Dali University]. https://doi.org/10.27811/d.cnki.gdixy.2025.000097

Xiong, W. (2024). *The current situation of self-awareness in junior high school students and practical research on teaching improvement* (in Chinese) [Master's thesis, Nanchang University]. https://doi.org/10.27232/d.cnki.gnchu.2024.000294

Xu, G. (2005). Research on the influence of emotional education on the emotional stability of first-year junior high school students (in Chinese). *Xinyu College Journal*, (6), 86–88.

Xu, P. (2017). *Social-emotional learning curriculum program for preschool children and its effectiveness research* (in Chinese) [Doctoral dissertation, Shanghai Normal University].

Yan, B. (2006). *Research on the influence of emotional intelligence cultivation on high school physics learning* (in Chinese) [Master's thesis, Fujian Normal University].

Yan, Z. (2014). *The relationship between emotional regulation ability, attitude towards academic burden, and mental health of eighth-grade students and intervention research* (in Chinese) [Master's thesis, Shaanxi Normal University].

Yang, H. (2024). *The influence of "life education" on "prosocial behavior" of upper-grade primary school students and intervention research* (in Chinese) [Master's thesis, Dali University]. https://doi.org/10.27811/d.cnki.gdixy.2024.000403

Yin, J. (2016). *Application research of social emotional education in reducing internalizing problem behaviors of middle class kindergarten children* (in Chinese) [Master's thesis, Shanghai Normal University].

Yu, H. (2022). *The relationship between cyberbullying behavior, peer relationships, and emotional regulation strategies in upper-grade primary school students* (in Chinese) [Master's thesis, Hebei North University]. https://doi.org/10.27767/d.cnki.ghbbf.2022.000312

Yuan, J. (2011). *Investigation on the current situation of cognitive emotional regulation ability of eighth-grade students and intervention research* (in Chinese) [Master's thesis, Hunan Normal University].

Zhang, L. (2015). *Preliminary practice of cultivating social emotional ability in junior high school students* (in Chinese) [Master's thesis, Shaanxi Normal University].

Zhang, Y. R. (2020). *An experimental study on the impact of social emotional learning on peer interaction ability of 4-6 year old children* (in Chinese) [Master's thesis, Shenyang Normal University]. https://doi.org/10.27328/d.cnki.gshsc.2020.000154

Zhao, L. (2022). *"Social emotional learning": International experience and localized practice research on campus bullying prevention* (in Chinese) [Master's thesis, Northwest Normal University]. https://doi.org/10.27410/d.cnki.gxbfu.2022.000654

Zheng, M. (2018). *The relationship between emotional intelligence and social anxiety in junior high school students and intervention research* (in Chinese) [Master's thesis, Hebei Normal University].

Zhu, Y. (2015). *The current situation of emotional regulation in boarding junior high school students and empirical research on curriculum intervention: Taking a boarding junior high school in urban Shanghai as an example* (in Chinese) [Master's thesis, Shanghai Normal University].

Table S4. Descriptive Cross-Tabulation of Grade Level and Other Characteristics

Grade Level	Overall	Preschool	Elementary	Secondary
**Methodological characteristics**				
Publication type				
Published article	9	1	4	4
Thesis	43	9	11	23
Research design				
RCT	2	1	0	1
QED	50	9	15	26
Sample size				
Small (<250)	46	10	11	25
Large (≥250)	6	0	4	2
Source of outcome data				
Student	41	1	14	26
Other	11	9	1	1
**Intervention features**				
Intervention approach				
Single-component	44	5	13	26
Comprehensive	8	5	2	1
Implementer				
Teacher	8	3	2	3
Researcher	26	6	9	11
Other	4	0	1	3
Unknown	14	1	3	10
**Recipients' characteristics**				
General area of school				
Rural	4	0	2	2
Suburban	1	0	1	0
Urban	47	10	12	25

https://doi.org/10.3390/bs16030368

Figure S1. Traffic Light Plot of Risk of Bias Assessments for the Included Studies Using the ROBINS-I Tool

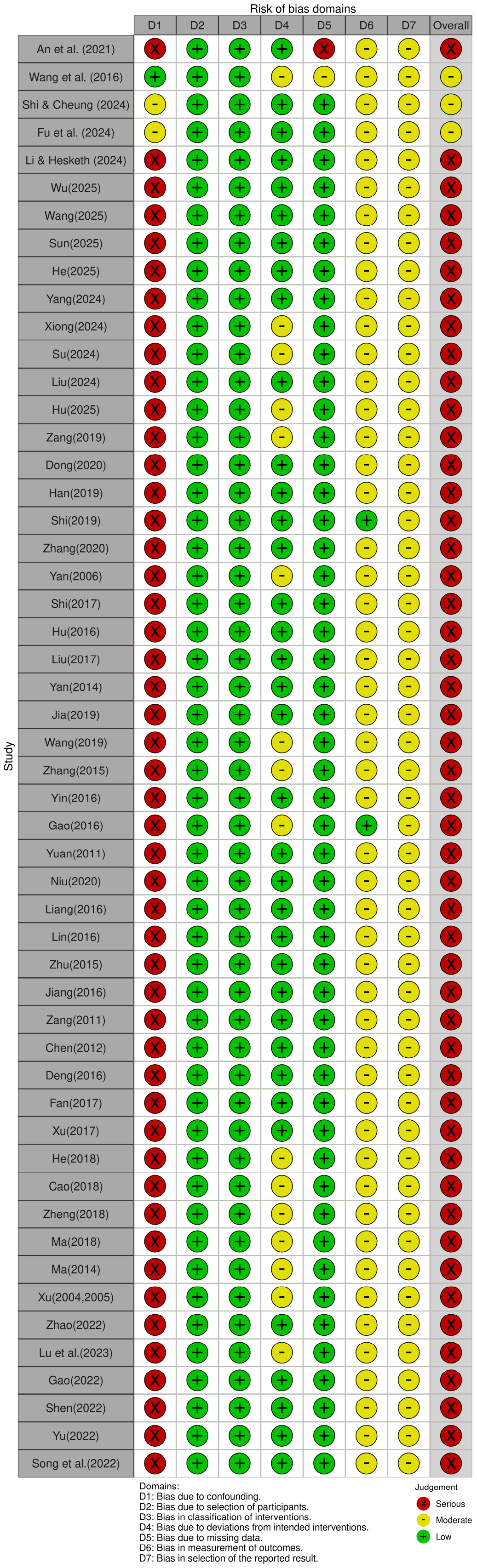

Figure S2. Forest Plot of the Effect Sizes

Meta Analysis

Study name	Outcome	Hedges's g	Standard error	Variance	Lower limit	Upper limit	Z-Value	p-Value
		Statistics for each study						
An et al. (2021)	EIQ-emotional intelligence	0.075	0.203	0.041	-0.323	0.473	0.369	0.712
Wang et al. (2016)	Combined	0.020	0.026	0.001	-0.031	0.071	0.772	0.440
Shi & Cheung (2024)	Combined	0.157	0.105	0.011	-0.048	0.362	1.504	0.133
Fu et al. (2024)	Combined	0.181	0.057	0.003	0.070	0.292	3.188	0.001
Li & Hesketh (2024)	Combined	0.072	0.091	0.008	-0.107	0.250	0.790	0.430
Wu (2025)	Combined	0.961	0.270	0.073	0.432	1.490	3.559	0.000
Wang (2025)	Combined	0.420	0.258	0.066	-0.085	0.926	1.631	0.103
Sun (2025)	Combined	0.513	0.213	0.045	0.097	0.930	2.415	0.016
He (2025)	Combined	0.758	0.227	0.052	0.313	1.203	3.337	0.001
Yang (2024)	Combined	0.432	0.134	0.018	0.169	0.695	3.218	0.001
Xiong (2024)	Combined	1.131	0.215	0.046	0.709	1.553	5.255	0.000
Su (2024)	Combined	0.482	0.195	0.038	0.100	0.864	2.471	0.013
Liu (2024)	Self-Efficacy for Emotion Regulation	0.953	0.206	0.042	0.550	1.356	4.635	0.000
Hu (2025)	Combined	0.459	0.219	0.048	0.029	0.889	2.094	0.036
Qi (2019)	Combined	0.229	0.152	0.023	-0.068	0.526	1.509	0.131
Dong (2020)	Combined	0.817	0.227	0.051	0.373	1.262	3.605	0.000
Han (2019)	Emotional Intelligence	1.033	0.197	0.039	0.648	1.419	5.254	0.000
Shi (2019)	Combined	0.813	0.281	0.079	0.262	1.363	2.894	0.004
Zhang (2020)	Peer Interaction Skills	1.195	0.155	0.024	0.891	1.500	7.693	0.000
Yan (2006)	Combined	0.511	0.202	0.041	0.115	0.907	2.530	0.011
Shi (2017)	Combined	0.466	0.211	0.045	0.052	0.880	2.206	0.027
Hu (2016)	Social Emotional Health	0.228	0.191	0.036	-0.146	0.602	1.197	0.231
Liu (2017)	Combined	0.392	0.188	0.035	0.024	0.761	2.085	0.037
Yan (2014)	Combined	0.003	0.139	0.019	-0.269	0.275	0.024	0.981
Jia (2019)	Emotional Intelligence	1.198	0.242	0.059	0.723	1.673	4.944	0.000
Wang (2019)	Combined	0.235	0.209	0.044	-0.175	0.644	1.124	0.261
Zhang (2015)	Antisocial Behavior	0.000	0.220	0.048	-0.431	0.431	0.000	1.000
Yin (2016)	Loneliness and Social Dissatisfaction	0.652	0.260	0.067	0.143	1.160	2.510	0.012
Gao (2016)	Relational Aggression	0.425	0.250	0.062	-0.065	0.915	1.700	0.089
Yuan (2011)	Combined	0.324	0.143	0.021	0.043	0.605	2.259	0.024
Niu (2020)	Combined	0.937	0.226	0.051	0.494	1.379	4.145	0.000
Liang (2016)	Combined	1.264	0.228	0.052	0.818	1.711	5.548	0.000
Lin (2016)	Combined	0.717	0.158	0.025	0.406	1.027	4.524	0.000
Zhu (2015)	Combined	0.316	0.255	0.065	-0.183	0.815	1.240	0.215
Jiang (2016)	Emotional Intelligence	0.339	0.142	0.020	0.061	0.617	2.387	0.017
Qi (2011)	Emotional Intelligence	0.516	0.198	0.039	0.128	0.904	2.606	0.009
Chen (2012)	Combined	0.129	0.162	0.026	-0.188	0.447	0.800	0.424
Deng (2016)	Emotional Intelligence	0.832	0.231	0.053	0.379	1.285	3.601	0.000
Fan (2017)	Emotional Intelligence	0.786	0.159	0.025	0.474	1.099	4.931	0.000
Xu (2017)	Combined	0.022	0.180	0.032	-0.331	0.374	0.120	0.905
He (2018)	Combined	0.309	0.102	0.010	0.110	0.508	3.046	0.002
Cao (2018)	Combined	0.309	0.152	0.023	0.010	0.607	2.026	0.043
Zheng (2018)	Combined	0.770	0.264	0.070	0.252	1.288	2.912	0.004
Ma (2018)	Emotional Intelligence	0.435	0.243	0.059	-0.041	0.911	1.792	0.073
Ma (2014)	Emotional Intelligence	0.525	0.225	0.051	0.083	0.966	2.330	0.020
Xu (2005)	Emotional Stability	0.814	0.206	0.042	0.411	1.217	3.956	0.000
Zhao (2022)	Combined	0.499	0.112	0.012	0.280	0.718	4.470	0.000
Lu et al. (2023)	Emotional Intelligence	2.730	0.269	0.072	2.203	3.257	10.149	0.000
Gao (2022)	Combined	1.007	0.304	0.093	0.410	1.603	3.306	0.001
Shen (2022)	Combined	0.163	0.155	0.024	-0.141	0.468	1.051	0.293
Yu (2022)	Combined	0.866	0.223	0.050	0.429	1.302	3.885	0.000
Song et al.(2022)	Peer Interaction Skills	1.165	0.155	0.024	0.862	1.469	7.529	0.000
		0.563	0.059	0.003	0.448	0.678	9.576	0.000

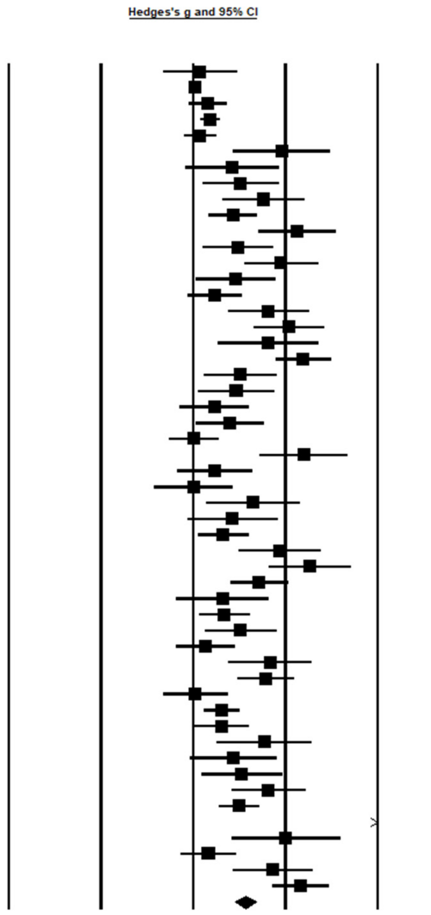

https://doi.org/10.3390/bs16030368
